# Supplementary material for: Inherently interpretable position-aware convolutional motif kernel networks for biological sequencing data
Source: Sci Rep. 2023 Oct 11;13:17216. doi: 10.1038/s41598-023-44175-7 (PMC10567796; doi:10.1038/s41598-023-44175-7)
Supplement: Supplementary file 2 — Supplementary Information 2. [file 41598_2023_44175_MOESM2_ESM.pdf]

# Inherently interpretable position-aware convolutional motif kernel networks for biological sequencing data
# Supplementary materials

## 1 Societal and Environmental Impact

Medical data are notoriously biased against minorities and there are numerous examples for machine learning models that learn this biases and have a severe deterioration in performance with regard to minorities (see e.g., [5]). We did not include a statement on the societal impact of our work into the main manuscript due to the used benchmarks. The HIV benchmark consists of viral sequences, which renders the evaluation regarding human metadata unnecessary. On the contrary, improving the treatment of HIV infection and AIDS has a beneficial impact on society. On the other hand, the splice site benchmarks are created using human genes. However, the original authors did not include any necessary metadata into the benchmarks, which renders a evaluation as described above impossible. We will include the evaluation of potential bias against minorities in future work, if the model is used in a real-world application on medical data that is potentially biased. Since this bias normally arises from the data, such considerations are task-specific and not model-specific.

All experiments were conducted using a single NVIDIA GeForce GTX 1080 Ti GPU. All experiments together required a total of 124 hours of computing time. This resulted in total emissions of 13.39 kg $CO_2$e, which is equivalent to burning 6.71 kg of coal. To compensate this emissions, 0.22 tree seedlings have to sequester carbon for 10 years. These estimations were calculated using the Machine Learning Impact calculator[1] by Lacoste and colleagues (Alexandre Lacoste et al.: *Quantifying the carbon emissions of machine learning.* arXiv preprint arXiv:1910.09700 (2019)).

## 2 Setting the Scaling Parameter

Given a sequence $\mathbf{x}$ with length $|\mathbf{x}|$, we found empirically that setting $\beta = \frac{|\mathbf{x}|^2}{10}$ compensates for the transformation of sequence positions as introduced in the manuscript (see Figure 1).

## 3 Normalized Position Frequency Matrix

A set of biological sequences of length $k$ can be easily transformed in to a position frequency matrix (PFM) by counting the occurrences of each nucleotide or amino acid from an alphabet $\mathbf{A}$ at each position and constructing a matrix $M = (m_{ij})_{i=1,\dots,|\mathbf{A}|;j=1,\dots,k}$, where $m_{ij}$ is equal to the number of occurrences of nucleotide or amino acid $i$ at position $j$. The PFM can then be easily transformed into a normalized position frequency matrix (nPFM) $M_{\text{norm}} = (m_{\text{norm},ij})_{i=1,\dots,|\mathbf{A}|;j=1,\dots,k}$ by dividing each entry of every column $m_j = \sum_i m_{ij}$ of $M$ by the $\ell_2$-norm of the respective column, i.e.

$$m_{\text{norm},ij} = \frac{m_{ij}}{\|m_j\|_2}. \tag{1}$$

Using this definition of an nPFM, the set of all $k$-mers is a subset of the set of all sequence motifs. Considering a k-mer as a special motifs, each column of the corresponding nPFM has only one element set to one while all other elements are set to zero.

[1] https://mlco2.github.io/impact/

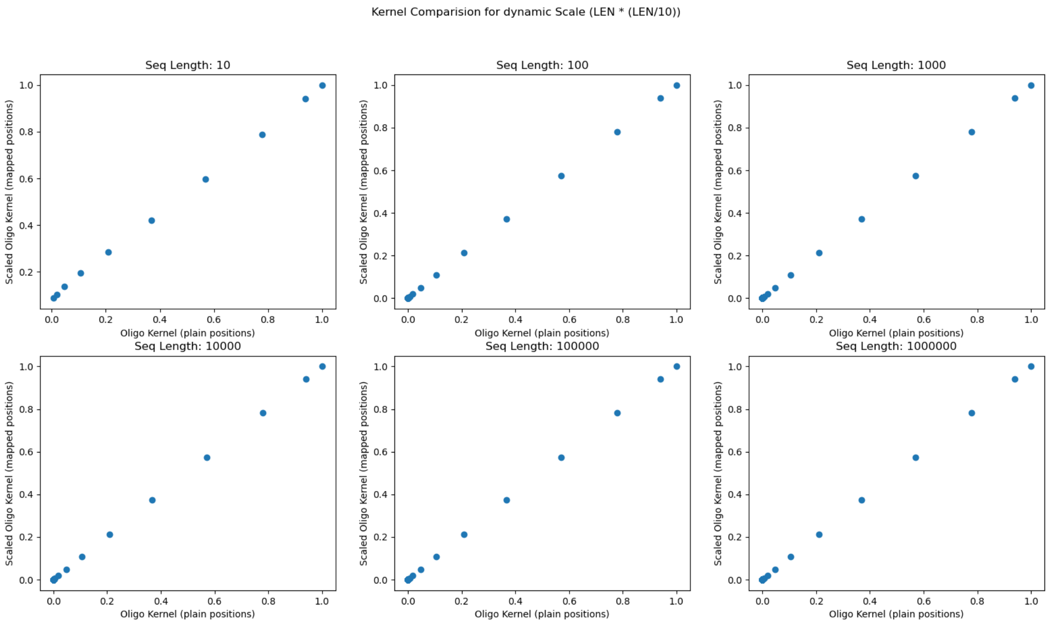


Figure 1: Comparison of the exponential term of the oligo kernel with the position kernel as introduced in the main manuscript in Eq. (1). Six different sequence lengths were tested: 10, 100, 1000, 10 000, 100 000, and 1 000 000. The scaling parameter $\beta$ of the position kernel was set to $\frac{|\mathbf{x}|^2}{10}$, where $|\mathbf{x}|$ denotes the corresponding sequence length. As noticeable in the plots, the chosen parameter value compensates for the reduced absolute distances of sequence positions and their dependence on the sequence length due to the projection onto the unit circle introduced in the main manuscript.

## 4 Orthogonal Projection onto the Subspace $\mathcal{E}$

We denote a motif position pair by $\mathbf{y} = (\omega, p)$. Then, the explicit parametrization derived by Mairal in [3] Appendix A also holds for our kernel. This can be easily shown by simple calculus using Mairal's definition:

$$f_{\mathbf{y}} := \sum_{j=1}^{n} \alpha_j^* \varphi(z_j) \qquad \text{with} \qquad \alpha^* \in \operatorname*{arg\,min}_{\alpha \in \mathbb{R}^n} \left\| \varphi(\mathbf{y}) - \sum_{j=1}^{n} \alpha_j \varphi(z_j) \right\|_{\mathcal{H}}^2 . \tag{2}$$

Then the following holds:

$$\begin{aligned} \left\| \varphi(\mathbf{y}) - \sum_{j=1}^{n} \alpha_j \varphi(z_j) \right\|_{\mathcal{H}}^2 &= \langle \varphi(\mathbf{y}), \varphi(\mathbf{y}) \rangle_{\mathcal{H}} - 2 \langle \varphi(\mathbf{y}), \sum_{j=1}^{n} \alpha_j \varphi(z_j) \rangle_{\mathcal{H}} + \langle \sum_{j=1}^{n} \alpha_j \varphi(z_j), \sum_{i=1}^{n} \alpha_i \varphi(z_i) \rangle_{\mathcal{H}} \\ &= 1 - 2 \sum_{j=1}^{n} \langle \varphi(\mathbf{y}), \alpha_j \varphi(z_j) \rangle_{\mathcal{H}} + \sum_{j=1}^{n} \sum_{i=1}^{n} \langle \alpha_j \varphi(z_j), \alpha_i \varphi(z_i) \rangle_{\mathcal{H}} \\ &= 1 - 2 \sum_{j=1}^{n} \alpha_j K_0(\mathbf{y}, z_j) + \sum_{j=1}^{n} \sum_{i=1}^{n} \alpha_j \alpha_i K_0(z_j, z_i) \\ &= 1 - 2\alpha^T K_Z(\mathbf{y}) + \alpha^T K_{ZZ} \alpha \end{aligned} \tag{3}$$

Here, $K_{ZZ} = (K_0(z_i, z_j))_{i=1,\ldots,n; j=1,\ldots,n}$ is the Gram matrix formed over the anchor points $z_1, \ldots, z_n$, $K_{ZZ}^{-\frac{1}{2}}$ is the (pseudo)-inverse square root of the Gram matrix, and $K_Z((\omega, p)) = (K_0(z_1, (\omega, p)), \ldots, K_0(z_n, (\omega, p)))^T$. $K_0$ is the kernel function introduced in the main manuscript. From here the argument is similar to Mairal's argument in the Appendix of [3].

## 5 Deriving the Position-Aware Motif Kernel from Motif Functions

For a sequence $\mathbf{x}$ over an alphabet $\mathbf{A}$ and a motif $\chi \in \mathbb{R}^{|\mathbf{A}|k}$, we define the motif function as

$$\phi_{\mathbf{x}}(\chi, t) = \sum_{p=1}^{|\mathbf{x}|} \phi_{\mathbf{x},p}(\chi, t), \tag{4}$$

with

$$\phi_{\mathbf{x},p}(\chi, t) = \exp\left(-\alpha \left\|\chi - \omega_p\right\|_2^2 - \frac{\beta}{2\sigma^2}\left\|t - \tilde{p}\right\|_2^2\right)$$

and position variable $t \in \mathbb{R}^2$.

Consider two sequences $\mathbf{x}$ and $\mathbf{x}'$ of same length, i.e. $|\mathbf{x}| = |\mathbf{x}'|$. The motif kernel between them is given as the inner product of the associated motif functions $\phi_{\mathbf{x}}$ and $\phi_{\mathbf{x}'}$:

$$\begin{aligned}
K_{\text{PAM}}(\mathbf{x}, \mathbf{x}') = \phi_{\mathbf{x}} \cdot \phi_{\mathbf{x}'} &= \int\int \phi_{\mathbf{x}}(\chi, t) \cdot \phi_{\mathbf{x}'}(\chi, t)\, d\chi\, dt \\
&= \sum_{p=1}^{|\mathbf{x}|}\sum_{q=1}^{|\mathbf{x}'|} \int\int e^{-\alpha\|\chi-\omega_p\|_2^2 - \frac{\beta}{2\sigma^2}\|t-\tilde{p}\|_2^2} \cdot e^{-\alpha\|\chi-\omega_q\|_2^2 - \frac{\beta}{2\sigma^2}\|t-\tilde{q}\|_2^2}\, d\chi\, dt \\
&= \sum_{p=1}^{|\mathbf{x}|}\sum_{q=1}^{|\mathbf{x}'|} \int\int e^{-\alpha\|\chi-\omega_p\|_2^2} \cdot e^{-\alpha\|\chi-\omega_q\|_2^2} \cdot e^{-\frac{\beta}{2\sigma^2}\|t-\tilde{p}\|_2^2} \cdot e^{-\frac{\beta}{2\sigma^2}\|t-\tilde{q}\|_2^2}\, d\chi\, dt \\
&= \sum_{p=1}^{|\mathbf{x}|}\sum_{q=1}^{|\mathbf{x}'|} \int e^{-\alpha\left(\|\chi-\omega_p\|_2^2 + \|\chi-\omega_q\|_2^2\right)}\, d\chi \cdot \int e^{-\frac{\beta}{2\sigma^2}\left(\|t-\tilde{p}\|_2^2 + \|t-\tilde{q}\|_2^2\right)}\, dt \\
&\overset{(6)}{=} \sum_{p=1}^{|\mathbf{x}|}\sum_{q=1}^{|\mathbf{x}'|} \sqrt{\frac{\pi}{2\alpha}} e^{-\frac{\alpha}{2}\|\omega_p-\omega_q\|_2^2} \sqrt{\frac{\pi\sigma^2}{\beta}} \cdot e^{-\frac{\beta}{4\sigma^2}\|\tilde{p}-\tilde{q}\|_2^2} \\
&\overset{(8)}{=} \sqrt{\frac{\pi^2\sigma^2}{2\alpha\beta}} \sum_{p=1}^{|\mathbf{x}|}\sum_{q=1}^{|\mathbf{x}'|} e^{\alpha\left(\omega_p^T\omega_q - k\right) + \frac{\beta}{2\sigma^2}\left(\tilde{p}^T\tilde{q} - 1\right)},
\end{aligned} \tag{5}$$

with $\omega_p, \omega_q \in \mathbb{R}_+^{|\mathbf{A}|k}$ and $\tilde{p}, \tilde{q}$ from the upper half of the unit circle. The above derivation uses the fact that the variables $\chi \in \mathbb{R}^{|\mathbf{A}|k}$ and $t \in \mathbb{R}^2$ are not restricted and thus (using the substitutions $y = \chi$, $\gamma = \alpha$, $\delta = \omega_p$, $\epsilon = \omega_q$,

$N = |\mathbf{A}|k$ and $y = t$, $\gamma = \frac{\beta}{2\sigma^2}$, $\delta = \tilde{p}$, $\epsilon = \tilde{q}$, $N = 2$, respectively) can be removed via

$$\begin{aligned}
&\int e^{-\gamma\left(\|y-\delta\|_2^2+\|y-\epsilon\|_2^2\right)}\,dy \\
&= \int_{-\infty}^{\infty}\cdots\int_{-\infty}^{\infty} e^{-\gamma\left((y_1-\delta_1)^2+\ldots+(y_N-\delta_N)^2+(y_1-\epsilon_1)^2+\ldots+(y_N-\epsilon_N)^2\right)}\,dy_1\ldots dy_N \\
&= \int_{-\infty}^{\infty} e^{-\gamma(y_1-\delta_1)^2} e^{-\gamma(y_1-\epsilon_1)^2}\,dy_1\cdot\ldots\cdot\int_{-\infty}^{\infty} e^{-\gamma(y_N-\delta_N)^2} e^{-\gamma(y_N-\epsilon_N)^2}\,dy_N \\
&= \int_{-\infty}^{\infty} e^{-\gamma\left((y_1-\delta_1)^2+(y_1-\epsilon_1)^2\right)}\,dy_1\cdot\ldots\cdot\int_{-\infty}^{\infty} e^{-\gamma\left((y_N-\delta_N)^2+(y_N-\epsilon_N)^2\right)}\,dy_N \\
&= \int_{-\infty}^{\infty} e^{-2\gamma y_1^2+2\gamma(\delta_1+\epsilon_1)y_1-\gamma(\delta_1^2+\epsilon_1^2)}\,dy_1\cdot\ldots\cdot\int_{-\infty}^{\infty} e^{-2\gamma y_N^2+2\gamma(\delta_N+\epsilon_N)y_N-\gamma(\delta_N^2+\epsilon_N^2)}\,dy_N \\
&\overset{(7)}{=} \sqrt{\frac{\pi}{2\gamma}} e^{\frac{(2\gamma(\delta_1+\epsilon_1))^2}{8\gamma}-\gamma(\delta_1^2+\epsilon_1^2)}\cdot\ldots\cdot\sqrt{\frac{\pi}{2\gamma}} e^{\frac{(2\gamma(\delta_N+\epsilon_N))^2}{8\gamma}-\gamma(\delta_N^2+\epsilon_N^2)} \\
&= \sqrt{\frac{\pi}{2\gamma}}\left(e^{\gamma\left(\frac{1}{2}(\delta_1^2+2\delta_1\epsilon_1+\epsilon_1^2)-\delta_1^2-\epsilon_1^2\right)}\cdot\ldots\cdot e^{\gamma\left(\frac{1}{2}(\delta_N^2+2\delta_N\epsilon_N+\epsilon_N^2)-\delta_N^2-\epsilon_N^2\right)}\right) \\
&= \sqrt{\frac{\pi}{2\gamma}}\left(e^{-\frac{\gamma}{2}\left(\delta_1^2-2\delta_1\epsilon_1+\epsilon_1^2\right)}\cdot\ldots\cdot e^{-\frac{\gamma}{2}\left(\delta_N^2-2\delta_N\epsilon_N+\epsilon_N^2\right)}\right) \\
&= \sqrt{\frac{\pi}{2\gamma}}\left(e^{-\frac{\gamma}{2}(\delta_1-\epsilon_1)^2}\cdot\ldots\cdot e^{-\frac{\gamma}{2}(\delta_N-\epsilon_N)^2}\right) \\
&= \sqrt{\frac{\pi}{2\gamma}} e^{-\frac{\gamma}{2}\|\delta-\epsilon\|_2^2}
\end{aligned} \tag{6}$$

using

$$\int_{-\infty}^{\infty} e^{-ay^2+by+c}\,dy = \sqrt{\frac{\pi}{a}} e^{\frac{b^2}{4a}+c} \tag{7}$$

with $a = 2\gamma$, $b = 2\gamma(\delta_i+\epsilon_i)$, and $c = -\gamma(\delta_i^2+\epsilon_i^2)$.

The last equality in Eq. (5) results from the normalisation of the nPFM as defined in Eq. (1), i.e., the column-wise unit $\ell_2$-norm demanded earlier in Section 5.1 of the main manuscript. Consider two normalized position frequency matrices (nPFM) $A \in \mathbb{R}_+^{|\mathbf{A}|\times k}$ and $B \in \mathbb{R}_+^{|\mathbf{A}|\times k}$, with $|\mathbf{A}|$ being the size of the alphabet over which the motif is created and $k$ being the length of the motif. One can define two vectors $a$ and $b$ given as flattened nPFMs $A$ and $B$, i.e., the columns are concatenated to convert the matrices into vectors. Utilizing that each column of a nPFM has unit $\ell_2$-norm by definition, the following equality is obtained:

$$\begin{aligned}
\|a-b\|^2 &= (a_{11}-b_{11})^2+\ldots+(a_{|A|1}-b_{|A|1})^2+\ldots+(a_{1k}-b_{1k})^2+\ldots+(a_{|A|k}-b_{|A|k})^2 \\
&= \underbrace{a_{11}^2+\ldots+a_{|A|1}^2}_{=1}+\underbrace{b_{11}^2+\ldots+b_{|A|1}^2}_{=1}+\ldots+\underbrace{a_{1k}^2+\ldots+a_{|A|k}^2}_{=1}+\underbrace{b_{1k}^2+\ldots+b_{|A|k}^2}_{=1} \\
&\quad -\underbrace{2(a_{11}b_{11}+\ldots+a_{|A|1}b_{|A|1}+\ldots+a_{1k}b_{1k}+\ldots+a_{|A|k}b_{|A|k})}_{=ab} \\
&= 2k-2ab \quad\Rightarrow\quad -\frac{1}{2}\|a-b\|^2 = ab-k
\end{aligned} \tag{8}$$

# 6 HIVdb

HIVdb is one of the largest, publicly available databases containing resistance information against antiretroviral drugs that are used for the treatment of an HIV infection or an acquired immunodeficiency syndrome (AIDS). We used available information for eight PI drugs, six NRTI drugs, and four NNRTI drugs (data was downloaded on 14 April 2021). The PI dataset contained 819 isolates, each represented by an amino acid sequence of length 99. The NRTI dataset contained 489 isolates, each represented by an amino acid sequence of length 240. Finally, the NNRTI dataset contained 583 isolates, each represented by an amino acid sequence of length 240. For each isolate, available drug resistance information for each drug of the corresponding type was given as a change in

Table 1: Overview of the HIVdb datasets. For each drug, basic dataset statistics are displayed as well as the best values for the positional uncertainty parameter $\sigma$ and the number of anchor points. Furthermore, mean values as well as standard deviations for four performance parameters (accuracy, F1 score, area under the receiver operating characteristic curve (auROC), and Matthew's correlation coefficient (MCC)) achieved on validation sets during a stratified 5-fold cross validation are shown.

| **Type** | **Drug** | **Samples** | **Class Distribution (%)** | | **Best Parameters** | | **Accuracy** | **F1 Score** | **auROC** | **MCC** |
|---|---|---|---|---|---|---|---|---|---|---|
| | | | susceptible | resistant | $\sigma$ | anchors | | | | |
| PI | | | | | | | | | | |
| | ATV | 477 | 59.3 | 40.7 | 16 | 50 | 0.918 ± 0.041 | 0.901 ± 0.051 | 0.969 ± 0.011 | 0.832 ± 0.086 |
| | DRV | 273 | 87.9 | 12.1 | 4 | 99 | 0.945 ± 0.033 | 0.779 ± 0.118 | 0.970 ± 0.019 | 0.757 ± 0.132 |
| | FPV | 737 | 65.3 | 34.7 | 8 | 99 | 0.912 ± 0.022 | 0.873 ± 0.032 | 0.965 ± 0.018 | 0.806 ± 0.049 |
| | IDV | 771 | 58.5 | 41.5 | 16 | 75 | 0.930 ± 0.019 | 0.915 ± 0.022 | 0.975 ± 0.008 | 0.856 ± 0.038 |
| | LPV | 612 | 67.2 | 32.8 | 4 | 50 | 0.920 ± 0.012 | 0.881 ± 0.017 | 0.970 ± 0.006 | 0.822 ± 0.026 |
| | NFV | 793 | 46.5 | 53.5 | 16 | 99 | 0.927 ± 0.014 | 0.932 ± 0.013 | 0.971 ± 0.008 | 0.853 ± 0.027 |
| | SQV | 775 | 63.4 | 36.6 | 8 | 99 | 0.939 ± 0.015 | 0.916 ± 0.022 | 0.980 ± 0.009 | 0.869 ± 0.033 |
| | TPV | 306 | 76.8 | 23.2 | 8 | 75 | 0.876 ± 0.044 | 0.725 ± 0.108 | 0.898 ± 0.032 | 0.646 ± 0.135 |
| NRTI | | | | | | | | | | |
| | 3TC | 477 | 45.3 | 54.7 | 16 | 120 | 0.937 ± 0.023 | 0.944 ± 0.023 | 0.973 ± 0.013 | 0.879 ± 0.049 |
| | ABC | 480 | 36.9 | 63.1 | 16 | 180 | 0.919 ± 0.017 | 0.935 ± 0.014 | 0.962 ± 0.017 | 0.828 ± 0.033 |
| | AZT | 477 | 58.5 | 41.5 | 16 | 120 | 0.899 ± 0.025 | 0.882 ± 0.026 | 0.965 ± 0.016 | 0.799 ± 0.047 |
| | D4T | 479 | 57.6 | 42.4 | 16 | 120 | 0.854 ± 0.045 | 0.826 ± 0.056 | 0.916 ± 0.030 | 0.701 ± 0.094 |
| | DDI | 479 | 52.0 | 48.0 | 16 | 240 | 0.862 ± 0.033 | 0.854 ± 0.038 | 0.914 ± 0.029 | 0.726 ± 0.066 |
| | TDF | 395 | 73.4 | 26.6 | 8 | 240 | 0.843 ± 0.034 | 0.711 ± 0.070 | 0.871 ± 0.050 | 0.609 ± 0.093 |
| NNRTI | | | | | | | | | | |
| | EFV | 511 | 55.4 | 44.6 | 16 | 180 | 0.924 ± 0.019 | 0.913 ± 0.023 | 0.965 ± 0.015 | 0.846 ± 0.039 |
| | ETR | 183 | 66.1 | 33.9 | 8 | 120 | 0.886 ± 0.043 | 0.829 ± 0.065 | 0.909 ± 0.061 | 0.750 ± 0.093 |
| | NVP | 515 | 47.0 | 53.0 | 16 | 120 | 0.920 ± 0.019 | 0.924 ± 0.019 | 0.966 ± 0.010 | 0.843 ± 0.036 |
| | RPV | 83 | 66.3 | 33.7 | | | *Removed from analysis due to low sample count* | | | |

fold resistance compared to the wildtype. We used thresholds provided by HIVdb to convert fold resistance values into the discrete classes 'susceptible' and 'resistant'.

HIVdb stores the isolate information as a tab-separated table. Each position is represented either by a '-', if the amino acid is the same as in the wild type, or by the mutation at that position. Furthermore, there are columns for each drug with the fold resistance of the isolate compared to the wild type. We provide a script that translates these tables into a FASTA file that can be used directly as input for the provided *DataSet* object. HIVdb provides two fold resistance thresholds for each of the drugs within the database. These thresholds are used by our preparation script to assign each isolate to either the 'low resistance', 'medium resistance', or 'high resistance' class. The *DataSet* object combines 'medium resistance' and 'high resistance' classes into the 'resistant' class on data access.

Table 1 provides detailed information about sample count, class distribution, best performing parameters, and classification performances for each of the tested drugs. The amount of isolates for each of the drugs differs from the total number of isolates, since not all isolates had fold resistance information for each of the drugs. We excluded drugs with less than 100 isolates from all further evaluations. Each of the drugs is indicated using the official three-letter abbreviation. The corresponding complete names and trade names are written below.

| **Type** | **Abbreviation** | **Name** | **Trade Name** |
|---|---|---|---|
| PI | | | |
| | ATV | Atazanavir | Reyataz, Evotaz, others |
| | DRV | Darunavir | Prezista, Prezcobix, others |
| | FPV | Fosamprenavir | Lexiva, Telzir |
| | IDV | Indinavir | Crixivan |
| | LPV | Lopinavir | - |
| | NFV | Nelfinavir | Viracept |
| | SQV | Saquinavir | Invirase, Fortovase |
| | TPV | Tipranavir | Aptivus |
| NRTI | | | |
| | 3TC | Lamivudine | Epivir, Epivir-HBV, Zeffix, others |
| | ABC | Abacavir | Ziagen, others |
| | AZT[2] | Azidothymidine | Retrovir, others |
| | D4T | Stavudine | Zerit |
| | DDI | Didanosine | Videx |
| | TDF | Tenofovir disoproxil | Viread, others |
| NNRTI | | | |
| | EFV | Efavirenz | Sustiva, Stocrin, others |
| | ETR | Etravirine | Intelence |
| | NVP | Nevirapine | Viramune |
| | RPV | Rilpivirine | Edurant, Rekambys |

## 7 Splice Site Benchmarks

The NN269 benchmark consists of 1324 true targets for both acceptor and donor datasets that were collected from 269 human genes. The acceptor sequences consist of 90 nucleotides with the splice site acceptor dimer AG at positions 69 to 70. The donor sequences consist of 15 nucleotides with the splice site donor dimer GT at positions 8 to 9. Furthermore, the benchmark includes 5553 acceptor decoys and 4922 donor decoys. Each decoy sequence has the splice site dimers (AG for acceptor sites, GT for donor sites) at the same positions as the true targets. The DGSplicer benchmark consists of 2380 true acceptor targets and 2379 true donor targets that were extracted from 462 multi-exon human genes. The acceptor sequences have a length of 36 nucleotides with the dimer AG at positions 26 to 27. The donor sequences have a length of 18 nucleotides with the dimer GT at positions 10 to 11. As with NN269, decoy sequences are included in the benchmark, i.e., 400314 pseudo acceptor sites and 282955 pseudo donor sites. Both benchmarks are split into training and test sets. The detailed statistics about the used benchmarks for the prediction of splice sites can be found in Table 2. To deal with the huge imbalance between positive and negative samples within the DGSplicer benchmark, we randomly under-sampled the negative samples in the training sets to achieve a class ratio of $\frac{N_p}{N_{rn}} = 0.25$, where $N_p$ is the number of samples in the positive class

Table 2: Overview of the benchmarks used to evaluate CMKN on the splice site prediction task.

| Benchmark | Type | Samples | Class Distribution (%) | | Best Parameters | | |
|---|---|---|---|---|---|---|---|
| | | | pos | neg | $k$ | $\sigma$ | anchors |
| NN269 | | | | | | | |
| | Acceptor (train) | 5788 | 19.3 | 80.7 | 2 | 16 | 45 |
| | Acceptor (test) | 1089 | 19.1 | 80.9 | | | |
| | Donor (train) | 5256 | 21.2 | 78.8 | 5 | 4 | 30 |
| | Donor (test) | 990 | 21.0 | 79.0 | | | |
| DGSplicer | | | | | | | |
| | Acceptor (train) | 322155 | 0.6 | 99.4 | 5 | 8 | 45 |
| | Acceptor (test) | 80539 | 0.6 | 99.4 | | | |
| | Donor (train) | 228267 | 0.8 | 99.2 | 5 | 4 | 45 |
| | Donor (test) | 57067 | 0.8 | 99.2 | | | |

and $N_{rn}$ is the number of samples in the negative class after resampling. Class ratios in validation and test sets were kept unchanged.

# 8 Hyperparameter Optimization

Experiments were conducted with Python 3.6.9 using imbalanced-learn 0.7.0, numpy 1.18.5, scikit-learn 0.23.2, scipy 1.5.2, and torch 1.6.0.

## 8.1 HIV Drug Resistance Prediction

### 8.1.1 CMKN

CMKN models have 5 hyperparameters associated with the motif kernel layer that need to be optimized. These include the k-mer length $k$, the motif comparison parameter $\alpha$, the position scaling parameter $\beta$, the positional uncertainty parameter $\sigma$, and the number of anchor points.

We used prior (biological) knowledge to fix three of the hyperparameters. This allowed us to reduce computation time and lower the $CO_2$ footprint of our experiments. We fixed the motif length $k$ to 1, since considering longer motifs offers no advantage in the case of resistance mutations against antiretroviral drugs. Previously conducted research suggest that a drug resistance is caused by exchanging single amino acids which justifies to set the motif length to 1. Additionally, we fixed $\alpha$ to 1.0 to ensure that the impact of inexact motif matching is *not* reduced. This takes the fact into account that some positions can have several different mutations causing resistance against antiretroviral drugs and the exchange of a single amino acid can cause a drug resistance. Therefore, inexact motif matching contains valuable information which justifies the value for $\alpha$ we used. Lastly, the position scaling parameter $\beta$ was fixed to $\frac{|\mathbf{x}|^2}{10}$, where $|\mathbf{x}|$ denotes the length of the input sequences. This value compensates for the transformation of the sequence positions as described in Section 2.

The other two hyperparameters, the positional uncertainty $\sigma$ and the number of anchor points, were optimized using a simple grid search. We used a small but sensible number of values for the grid to keep the $CO_2$ footprint of our experiments as low as possible. The following values were used for the grid search:

$$\sigma \in \{1, 2, 4, 8, 16\}$$
$$\#\text{anchors}_{\text{PI}} \in \{50, 75, 99\}$$
$$\#\text{anchors}_{\text{NRTI}} \in \{120, 180, 240\}$$
$$\#\text{anchors}_{\text{NNRTI}} \in \{120, 180, 240\}$$

The different choices for the number of anchors for PI and NRTI/NNRTI drugs were chosen to take account of the different sequence lengths. The optimal parameter choice for each of the drugs can be found in Table 1.

All CMKN models were trained using PyTorch's implementation of the ADAM algorithm and class-balanced loss

as introduced in [1]. Training lasted for 200 epochs and no early-stopping was used. The learning rate started at 0.1 and was dynamically adjusted using the *ReduceLROnPlateau* method.

The value combination that maximized the most of the four performance measures, accuracy, F1 score, auROC, and MCC, was selected for further analysis.

### 8.1.2 Models used for Comparison

We used standard parameters for the random forest classifiers trained on our HIVdb datasets that matched the parameters used in [6]. The number of trees was set to 500 while the number of features to look at, when searching for the best split, was set to the square root of the total number of features. For all other parameters the default values of the *sklearn.ensemble.RandomForestClassifier* class were used. This is coherent with the training procedure used in [6]. The input sequences were ordinal encoded before passing them to the classifier, i.e., each letter was replaced by an integer reflecting the position of the letter in the alphabet.

For support vector machines (SVMs) with polynomial kernel, we performed a grid search to optimize the degree of the polynomial kernel and the regularization parameter. The grid consisted of the following values:

$$\begin{aligned} \mathrm{C} &\in \{10^{-5}, 10^{-4}, 10^{-3}, 10^{-2}, 10^{-1}, 1, 10\} \\ \mathrm{degree} &\in \{1, 2, 3, 4, 5\} \end{aligned}$$

Similar to the CMKN models, the parameter combination that maximized the most of the four performance measures, accuracy, F1 score, auROC, and MCC, was selected for further analysis. We used the same ordinal encoding that was used in the RF experiment to encode the input for the SVMs. For the model selection of SVMs with oligo kernel, we used the input encoding as described in [4]. The hyperparameters of this method are the length of $k$-mers denoted by $k$, the position uncertainty parameter $\sigma$, and the regularization parameter C. Similar to CMKNs, $k$ was fixed to 1 due to biological reasons. The other two hyperparameter were optimized with the following grid:

$$\begin{aligned} \mathrm{C} &\in \{10^{-5}, 10^{-4}, 10^{-3}, 10^{-2}, 10^{-1}, 1, 10\} \\ \sigma &\in \{1, 2, 4, 8, 16\} \end{aligned}$$

The optimal parameters for the SVM models were:

| **Drug** | $\mathrm{SVM}_{\mathrm{poly}}$ | | $\mathrm{SVM}_{\mathrm{oligo}}$ | |
|---|---|---|---|---|
| | C | degree | C | $\sigma$ |
| ATV | 1.0 | 3 | 0.1 | 4 |
| DRV | 0.001 | 1 | 0.01 | 1 |
| FPV | 0.1 | 5 | 1.0 | 1 |
| IDV | 10.0 | 2 | 1.0 | 1 |
| LPV | 1.0 | 3 | 1.0 | 1 |
| NFV | 10.0 | 2 | 1.0 | 1 |
| SQV | 1.0 | 3 | 1.0 | 1 |
| TPV | 0.001 | 2 | 0.1 | 1 |
| 3TC | 0.1 | 4 | 0.1 | 1 |
| ABC | 0.1 | 4 | 0.1 | 1 |
| AZT | 0.1 | 5 | 1.0 | 1 |
| D4T | 0.1 | 3 | 0.1 | 1 |
| DDI | 0.1 | 3 | 0.1 | 1 |
| TDF | 0.01 | 5 | 1.0 | 1 |
| EFV | 0.01 | 5 | 0.1 | 2 |
| ETR | 0.1 | 4 | 10.0 | 4 |
| NVP | 10.0 | 1 | 0.1 | 4 |
| RPV | *Removed from analysis due to low sample count* | | | |

The CNN architecture used for DRM coverage comparison in section 9 was first published in [7]. First, we tried to train CNN models using the R script provided by the authors on our datasets but, unfortunately, the script exited with errors and was not usable. Instead of debugging the provided script, which would already pose the risk of changing the training environment compared to the original publication, we decided to implement the same architecture using PyTorch. This course of action provided the additional benefit that the training of both neural network architectures, CNN and CMKN, used the same framework and followed the same standards which improves comparability of the methods. The first layer was an embedding layer that took the nominal encoded sequences and learned a three-dimensional embedding. Afterwards two one-dimensional convolutional layers with ReLu activation were used. Both convolutional layers had a kernel size of 9 with 32 filters. A one-dimensional MaxPooling layer with a pooling size of 5 was used between the two convolutional layers. The flatted output of the second convolutional layer was fed into fully-connected layers for the classification. CNN models were trained using PyTorch's implementation of the ADAM algorithm and class-balanced loss as introduced in [1]. Training lasted for 200 epochs and no early-stopping was used. The learning rate started at 0.1 and was dynamically adjusted using the *ReduceLROnPlateau* method. We did not include the performance of the CNN architecture by Steiner et al. into our main manuscript due to the non-competitive performance on our datasets (i.e., they reached a mean MCC of 0.1). However, we included the description here since Steiner's models are used in section 9. The CNN results shown in the main manuscript were achieved with a network resulting from a simple ablation test, i.e., we replaced the kernel layer of an CMKN model with a standard convolutional layer with the same parameters.

### 8.2 Splice Site Prediciton

#### 8.2.1 CMKN

In contrast to the HIV experiments, we only fixed two hyperparameters in the CMKN models used for splice site prediction: $\alpha$ was fixed to 1 and $\beta$ was fixed as described in Section 2. The other hyperparameters were optimized with a grid search using the following values:

$$k \in \{2,3,4,5\}$$
$$\sigma \in \{1,2,4,8,16\}$$
$$\#\text{anchors} \in \{15,30,45\}$$

The optimal parameter choice for each splice site dataset can be found in Table 2.

All CMKN models were trained using PyTorch's implementation of the ADAM algorithm and class-balanced loss as introduced in [1]. Training lasted for 200 epochs and no early-stopping was used. The learning rate started at 0.1 and was dynamically adjusted using the *ReduceLROnPlateau* method.

## 9 Comparing the Interpretation Capabilities of CMKNs

Comparing the quality of interpretation capabilities of different methods is a difficult problem, due to the ethical and philosophical aspects of interpretation which are hard to express mathematically [2, 5]. Nevertheless, we performed some quantitative comparisons of the global interpretation between the used inherently interpretable methods (i.e., CMKNs and oligo kernel SVMs) as well as published *post-hoc* interpretations of non-interpretable methods (impurity-based feature importance for RFs [6] and permutation feature importance analysis for CNNs [7]) on the HIV prediction task. For all methods, the 20 most important positions were identified and the percental coverage of drug resistance mutation positions (DRMs) using these 20 positions was calculated. The results can be found in Figure 2. We found that CMKNs performed similar to *post-hoc* methods applied on CNNs and RFs when used to identify DRMs. All three methods significantly outperformed oligo kernel SVMs. However, the *post-hoc* methods were limited to identifying positions without compositional information of the mutations. That lead to an incomplete picture of the biological process and therefore decreases the utility of the interpretation provided by *post-hoc* methods. Only CMKNs and oligo kernel SVMs were able to provide the complete picture with positional and compositional information of the drug resistance mutations. We used the procedure described by Meinicke and colleagues in the original manuscript to visualize the learned oligo functions from the trained SVMs [4]. The result is presented in Figure 3. Due to the fact that the oligo kernel is limited to discrete $k$-mers, the compositional variability of DRMs is not identified by oligo kernel SVMs. For identified DRMS, the visualization focuses on single amino acids.

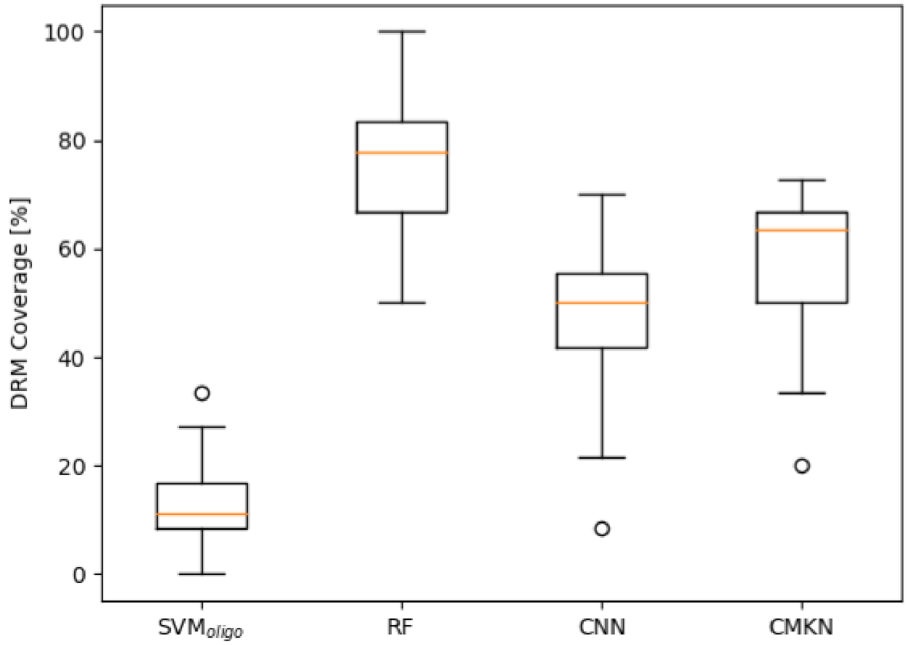


Figure 2: Number of identified drug resistance mutation positions (DRMs) by different models. *Post-hoc* methods were needed to calculate feature importances for some models: For convolutional neural network (CNN) classifiers, we applied a permutation feature importance analysis and for the random forest (RF) classifiers, we calculated impurity-based feature importances. Position importances were directly assessable for CMKNs and oligo kernel SVMs ($SVM_{oligo}$). CMKNs performed similar to CNNs and RFs. All three significantly outperformed $SVM_{oligo}$. The center line of each box indicates the median. The height of the boxes represents the inter quartile range (IQR) with the upper and lower whiskers set to 1.5 times the IQR. Outliers are depicted by circles.

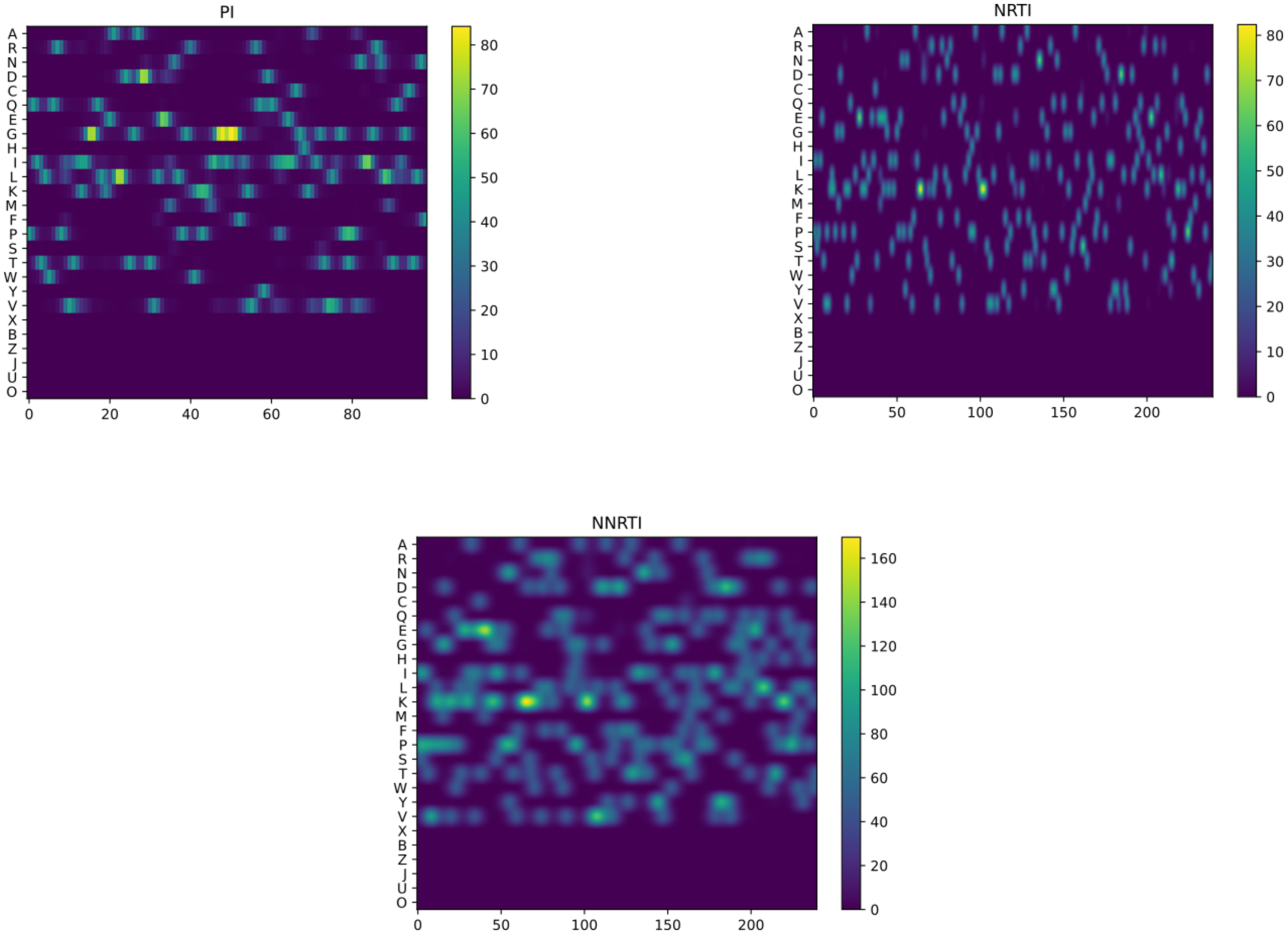


Figure 3: Interpretation of the support vector machines utilizing the oligo kernel ($SVM_{oligo}$). Details about the calculation of these matrices can be found in the original manuscript [4]. $SVM_{oligo}$ had difficulties identifying drug resistance mutation positions (DRMs) and, due to it's limitation to $k$-mers, focused on single amino acids for each DRM.

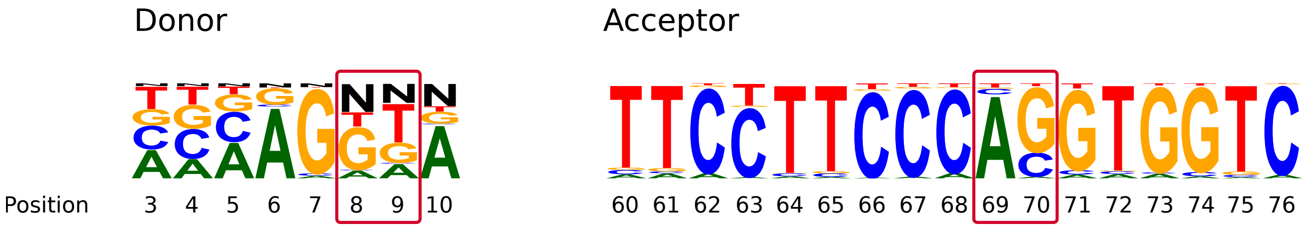


Figure 4: The learned sequence motifs of a CMKN model trained on the NN269 splice site prediction benchmark. The left motif was learned on the donor dataset. The right motif was learned on the acceptor dataset. The splice site is indicated by a red box.

Additionally to global interpretations, CMKN models can also provide local interpretations. Comparing local interpretations cannot be achieved without a user study, since their utility is closely coupled with the intended user. Performing a user study with medical practitioners is a time consuming task, which is out of the scope of this manuscript. Nevertheless, evaluating local interpretations with the intended users is an important research question and will be investigated in future work.

## 10 CMKN Model Interpretability for Splice Site Prediction

We also analyzed how well CMKN models can retrieve the relevant motifs from the splice site prediction task. For this purpose, we retrained a model on the two NN269 training sets (donor and acceptor). We selected motif lengths that are able to capture the compositional variability that contributes to real splice sites in contrast to the decoy sequences. Therefore, the model trained on the donor dataset used a motif length of 8 and the model trained on the acceptor dataset used a motif length of 17. Since there is no positional variability in the splice site benchmarks, we only investigated the learned motifs. Figure 4 shows the motifs learned on the donor (left) and acceptor (right) dataset. Both splice site dimers (GT for donor sites and AG for acceptor sites) are correctly learned by CMKN models. Note that a high compositional variability in the learned motifs for these two positions is expected since they are exactly the same in real splice site sequences and decoy sequences. Therefore, the model does not put high distinction into these two positions resulting in less clear motifs. For the donor sequences, CMKN is able to identify the AG dimer that appears directly before the splice site and is highly predicative for a real splice site sequence. For the acceptor sequences, CMKN correctly learns the poly pyrimidine tract that precedes a real splice site together with the fact that a guanine directly after the splice site dimer is highly predicative for a real splice site. The learned motifs show that CMKN models are able to recover biologically meaningful sequence motifs from datasets.

### References


[1] Y. Cui, M. Jia, T.-Y. Lin, Y. Song, and S. Belongie. Class-balanced loss based on effective number of samples. In *Proceedings of the IEEE Conference on Computer Vision and Pattern Recognition*, pages 9268–9277, 2019.

[2] Z. C. Lipton. The mythos of model interpretability: In machine learning, the concept of interpretability is both important and slippery. *Queue*, 16(3):31–57, 2018.

[3] J. Mairal. End-to-end kernel learning with supervised convolutional kernel networks. In *Advances in neural information processing systems*, pages 1399–1407, 2016.

[4] P. Meinicke, M. Tech, B. Morgenstern, and R. Merkl. Oligo kernels for datamining on biological sequences: a case study on prokaryotic translation initiation sites. *BMC bioinformatics*, 5(1):169, 2004.

[5] J. Morley, C. C. Machado, C. Burr, J. Cowls, I. Joshi, M. Taddeo, and L. Floridi. The ethics of ai in health care: a mapping review. *Social Science & Medicine*, 260:113172, 2020.

[6] L. M. Raposo, P. T. C. Rosa, and F. F. Nobre. Random forest algorithm for prediction of hiv drug resistance. In *Pattern Recognition Techniques Applied to Biomedical Problems*, pages 109–127. Springer, 2020.

[7] M. C. Steiner, K. M. Gibson, and K. A. Crandall. Drug resistance prediction using deep learning techniques on hiv-1 sequence data. *Viruses*, 12(5):560, 2020.
